# TAG‐SPARK: Empowering High‐Speed Volumetric Imaging With Deep Learning and Spatial Redundancy

**DOI:** 10.1002/advs.202405293

**Published:** 2024-09-16

**Authors:** Yin‐Tzu Hsieh, Kai‐Chun Jhan, Jye‐Chang Lee, Guan‐Jie Huang, Chang‐Ling Chung, Wun‐Ci Chen, Ting‐Chen Chang, Bi‐Chang Chen, Ming‐Kai Pan, Shun‐Chi Wu, Shi‐Wei Chu

**Affiliations:** ^1^ Graduate Institute of Electronics Engineering National Taiwan University Taipei 10617 Taiwan; ^2^ Department of Engineering and System Science National Tsing Hua University Hsinchu 30013 Taiwan; ^3^ Molecular Imaging Center National Taiwan University Taipei 10617 Taiwan; ^4^ Department of Physics National Taiwan University Taipei 10617 Taiwan; ^5^ Research Center for Applied Sciences (RCAS) Academia Sinica Taipei 115 Taiwan; ^6^ Department of Medical Research National Taiwan University Hospital Taipei 10002 Taiwan; ^7^ Department and Graduate Institute of Pharmacology National Taiwan University College of Medicine Taipei 10002 Taiwan; ^8^ Brain Research Center National Tsing Hua University Hsinchu 30013 Taiwan; ^9^ Institute of Biomedical Sciences Academia Sinica Taipei 11529 Taiwan; ^10^ Cerebellar Research Center National Taiwan University Hospital Yun‐Lin Branch Yun‐Lin 64041 Taiwan

**Keywords:** deep‐learning noise reduction, high‐speed volumetric image, neural networks, Purkinje cells, two‐photon microscopy

## Abstract

Two‐photon high‐speed fluorescence calcium imaging stands as a mainstream technique in neuroscience for capturing neural activities with high spatiotemporal resolution. However, challenges arise from the inherent tradeoff between acquisition speed and image quality, grappling with a low signal‐to‐noise ratio (SNR) due to limited signal photon flux. Here, a contrast‐enhanced video‐rate volumetric system, integrating a tunable acoustic gradient (TAG) lens‐based high‐speed microscopy with a TAG‐SPARK denoising algorithm is demonstrated. The former facilitates high‐speed dense z‐sampling at sub‐micrometer‐scale intervals, allowing the latter to exploit the spatial redundancy of z‐slices for self‐supervised model training. This spatial redundancy‐based approach, tailored for 4D (xyzt) dataset, not only achieves >700% SNR enhancement but also retains fast‐spiking functional profiles of neuronal activities. High‐speed plus high‐quality images are exemplified by in vivo Purkinje cells calcium observation, revealing intriguing dendritic‐to‐somatic signal convolution, i.e., similar dendritic signals lead to reverse somatic responses. This tailored technique allows for capturing neuronal activities with high SNR, thus advancing the fundamental comprehension of neuronal transduction pathways within 3D neuronal architecture.

## Graphical abstract

Leveraging the high‐speed dense z‐sampling at sub‐micrometer‐scale intervals of a tunable acoustic gradient (TAG) lens, a self‐supervised TAG‐SPARK denoising algorithm that exploits the spatial redundancy of z‐slices to enable >700% SNR enhancement of two‐photon video‐rate volumetric imaging while retaining fast‐spiking functional profiles of neuronal activities, and revealing intriguing dendritic‐to‐somatic signal convolution in Purkinje cells is developed.

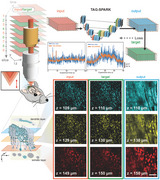

## 1 Introduction

The comprehensive recording of neural activities among large neuronal populations with high spatiotemporal resolution is of fundamental significance to understanding neural circuit signal transduction, information processing, and generation of behaviors, i.e., toward recognizing how the brain works through probing emergent functionality.^[^ ^1^ ^]^

Two‐photon microscopy (2PM) not only shows promise for simultaneously recording hundreds of neurons with sub‐micrometer spatial resolution but also provides optical sectioning capabilities for 3D brain recording.^[^ ^2^ ^]^ To directly observe neuronal activity dynamics, high temporal resolution is necessary. Although recent advancements in 2PM have improved imaging speed up to kHz frame rates, the observations are restricted to a 2D lateral plane.^[^ ^3^ ^]^ Given that neuronal networks are intrinsically distributed in 3D, several high‐speed volumetric 2PM schemes have been developed,^[^ ^4^ ^]^ including multi‐plane imaging,^[^ ^5^ ^]^ stereoscopic illumination with extended depth‐of‐field (DOF),^[^ ^6^ ^]^ and fast z‐focus modulation mechanisms.^[^ ^7^ ^]^ Most multi‐plane imaging strategies only offer a relatively low number of layers (N < 10) for volumetric imaging and the approach of extending DOF results in compromised accuracy when retrieving the accurate z‐position of structures. Alternatively, fast focus control techniques such as deformable mirrors^[^ ^8^ ^]^ and tunable acoustic gradient (TAG) lenses^[^ ^9^ ^]^ are able to acquire the full 3D information in detail. TAG lenses, for example, are capable of axially scanning and sampling hundreds of layers during volumetric imaging within a single transverse scan. Although these approaches provide sufficient volume rates for capturing neuronal activities, they usually suffer from a low SNR issue due to limited photon flux per voxel. High‐intensity illumination helps to improve image quality, but concurrent photobleaching, phototoxicity, and tissue damage restrict the applications of chronic monitoring.

Apart from optical approaches, image post‐processing provides an alternative solution to enhance SNR. In particular, deep‐learning methods, which harness the power of artificial neural networks accompanied by unsupervised or supervised learning, have demonstrated remarkable proficiency in handling complex patterns, making them applicable for tasks such as noise reduction and feature extraction.^[^ ^10^ ^]^ However, most unsupervised learning using patch‐based algorithms requires substantial effort in clustering the overlapping patches of noisy images with comparable noise levels prior to model training, posing a challenge for intricate image‐denoising tasks. For supervised learning, acquiring fast, high‐SNR dynamic neuron functional images as ground truth remains challenging. On the other hand, deep self‐supervised learning techniques present a promising avenue for addressing this challenge. Self‐supervised learning operates on the principle of exploiting inherent redundancies presented in the data, either in the spatial or temporal domain. Contemporary techniques leverage temporal information as the primary source for self‐supervised learning via the U‐Net structure. With the advance of high‐speed scanning technologies, acquiring datasets rich in temporal information has become accessible. Notable examples in the field of neural imaging, such as DeepCAD‐RT^[^ ^11^ ^]^ and DeepVID^[^ ^12^ ^]^ demonstrate the efficacy of utilizing consecutive image frames in time series as a training dataset to enhance the SNR. However, the temporal domain methods might introduce artifacts in the denoised output due to mixing information from neighboring time points, especially when tracing neuronal dynamics under high‐speed low‐contrast acquisition.

To avoid temporal artifacts, the idea of combining U‐Net and convolutional neural networks (CNN) was recently proposed as a statistically unbiased prediction utilizing spatiotemporal information in imaging data (SUPPORT).^[^ ^13^ ^]^ SUPPORT has successfully retained the dynamic functional responses compared to DeepCAD‐RT, which follows the Noise2Noise concept^[^ ^14^ ^]^ with temporal redundancy. However, both SUPPORT and conventional Noise2Noise are based on a 2D U‐Net model that identifies xyt or xyz images, but not volume functional images (xyzt).

Extending the concept of Noise2Noise, we have developed a noise reduction kernel named TAG‐SPARK (TAG‐lens‐based SPAtial redundancy‐driven noise Reduction Kernel), a 3D U‐Net‐based deep‐learning denoising model. Different from conventional Noise2Noise, which assumes input‐target pairs are independent and processes them individually, TAG‐SPARK creates two separate volume stacks as input‐target pairs with millisecond temporal dynamics, i.e., the dataset is intrinsically 4D. This approach allows us to exploit information across individual volumes using 3 × 3 × 3 convolution kernels, which ensures that neuronal temporal responses are preserved without distortion. The loss function used in Noise2Noise is the L2 norm, while TAG‐SPARK combines L1 and L2 norms to balance resilience to outliers and convergence. Thus, TAG‐SPARK and Noise2Noise differ significantly in input‐target pair formation, network architectures, and loss functions.

To the best of our knowledge, previously reported methods, such as DeepCAD‐RT, DeepVID, and SUPPORT, neither have used axial redundancy to enhance the signal‐to‐noise ratio through deep learning nor have applied the deep learning model to demonstrate volumetric functional imaging. Here, we present a high‐speed volumetric 2PM imaging system incorporated with a TAG lens, which enables dense z‐sampling of the brain tissue with a sub‐micrometer‐scale interval between layers. Leveraging the spatial redundancy inherent in this system, TAG‐SPARK offers more than 700% SNR enhancement of the acquired images. The combination of the TAG‐lens‐based volumetric system and TAG‐SPARK provides complementary advantages since the former allows high‐density‐in‐z volumetric mapping, whose inter‐layer structural similarity leads to the validity of the algorithm. Unlike conventional self‐supervised learning based on temporal redundancy, TAG‐SPARK is specifically trained by the spatial redundancy of the high‐density z‐sections in the 3D TAG‐based volumetric images. The 3D U‐Net‐based TAG‐SPARK is tailor‐made to simultaneously process the 4D functional data, i.e., 3D in space and 1D in time. As a consequence, our method manifests the ability to capture neuronal activities during animal behaviors without contaminating the temporal dynamics. In this work, we exemplify a proof‐of‐concept experiment via recording 3D calcium dynamics of in vivo Purkinje cells (PCs), which exhibit distinct temporal responses from tree‐like dendrites to spherical soma at various depths during signal transmission. Moreover, we found that similar dendritic signals lead to distinct somatic responses, manifesting the significance of the deep‐learning enhanced high‐speed two‐photon volumetric imaging system.

## 2 Results

### 2.1 TAG‐Lens‐Based High‐Speed 2PM Facilitating TAG‐SPARK

**Figure 1:**
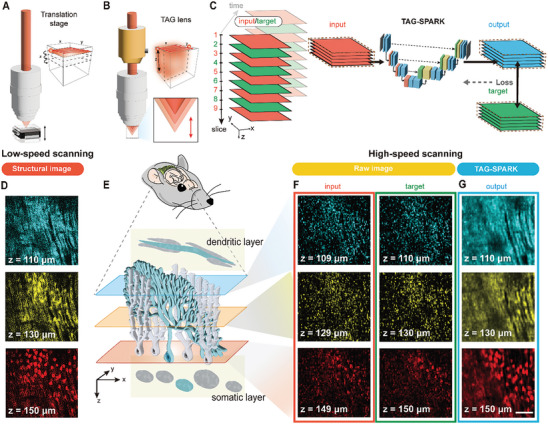
A high‐speed two‐photon volumetric system with TAG‐SPARK denoising algorithm. A) Schematic of low‐speed scanning mode for obtaining volumetric images utilizing a linear translation stage. B) Schematic of high‐speed scanning mode, where fast axial scanning is achieved by a TAG lens. C) Schematic of the TAG‐SPARK denoising network. Two adjacent z‐slices are taken as input‐target pairs for model training. The loss function (output/target) is used to optimize the network parameters. (D) Low‐speed structural images of PCs at different depths. E) Schematic of PCs from the dendritic layer to the somatic layer, showing the convolutional tree structure. In the xy sagittal planes, the dendrite branches exhibit parallel line structures, while the cell bodies display circular images. F, G) In vivo calcium imaging of PCs at different depths of high‐speed (F) and denoised high‐speed (G) scanning mode, employing the TAG‐SPARK model, respectively. Scale bars, 100 µm.

We constructed a high‐speed volumetric 2PM system with the primary objective of observing 3D neuronal dynamics within a living brain (Figure 4 in Materials and Methods). Our system allows for volumetric imaging using both low‐ and high‐speed z‐scanning modalities (**Figure** **1**A,B), where axial scanning was achieved through a linear translation stage and a TAG lens, respectively. Low‐speed scanning mode excelled at providing detailed structural mapping with high SNR, while high‐speed scanning mode was optimized for the interrogation of functional information. In our high‐speed configuration, a TAG lens enables a rapid ≈200 kHz axial scanning rate and a volume rate of 23 Hz. Moreover, a high‐speed data acquisition board allows dense z‐sampling of brain tissues below micrometer‐scale intervals. Remarkably, the voxel dwell times of the two modes are correspondingly 5 and 0.02 µs, featuring more than two orders of magnitude speed difference. While the TAG‐lens‐based high‐speed 2PM provides an adequate volume rate to monitor calcium responses, the short voxel dwell time poses challenges in achieving satisfactory SNR due to limited signal photon flux and stochastic noise coupling.

To address this issue, we developed TAG‐SPARK, a deep‐learning model based on the 3D U‐Net architecture, as illustrated in Figure 1C. TAG‐SPARK is trained in a spatial manner, leveraging the spatial redundancy in TAG lens z‐sampling within each volumetric dataset. This approach involves adjacent z‐slices as input‐target pairs for model training. Following the Noise2Noise concept, one major advantage of TAG‐SPARK is that ground‐truth measurement accumulation is no longer necessary. The fundamental concept is that these adjacent input‐target pairs shall exhibit a comparable image context but are contaminated by independent noises. As a result, each iteration allows for the refinement of network parameters to achieve convergence of the loss function, which, in turn, enables noise reduction and the extraction of the distinctive morphology. The loss function is determined by comparing the output with the target. Notably, as the model training is accomplished independently within each volume, the denoising process does not influence the signal strength in other volumes, leaving no artifact for temporal dynamics.

The TAG‐SPARK volumetric 2PM system was applied for the examination of a specific neural structure, mouse PCs, known for their tree‐like dendritic structures and the fast dynamics of dendritic calcium spikes.^[^ ^15^ ^]^ The PCs were labeled with GCaMP to observe the two‐photon calcium dynamics. Sample preparation was described in Materials and Methods. The low‐speed scanning mode generated detailed structural mapping with high SNR as shown in Figure 1D. At a depth of 110 µm, parallel line structures revealed dendrite branches in distinct sagittal planes. As the depth reaches 130 µm, dendrite branches converged into circular cell bodies (soma), consistent with the typical spacing between dendrite and soma in PCs.^[^ ^16^ ^]^ At the bottom layer, the cell bodies of PCs exhibited flask‐like shapes. The overall z‐section images matched the well‐known structure of PCs in the cerebellar cortex as shown in Figure 1E. Regarding the high‐speed scanning mode, dense z‐sampling with a TAG lens satisfies the Nyquist sampling theorem. This allowed for adjacent z‐slices to contain similar biological contexts, facilitating TAG‐SPARK model training. Figure 1F displayed high‐speed imaging examples at three distinct depths with adjacent pairs (109 and 110 µm; 129 and 130 µm; 149 and 150 µm), presenting the similarity in each pair. Nevertheless, low SNR made it challenging to distinguish the PCs neuronal architecture. Our TAG‐SPARK image reconstruction process significantly reduced the noise contamination and enhanced the contrast of the high‐speed scanning image, retrieving individual dendritic and somatic structures of PCs, as exemplified in Figure 1G.

### 2.2 Spatial and Temporal Characterization of the Volumetric TAG‐SPARK Imaging

**Figure 2:**
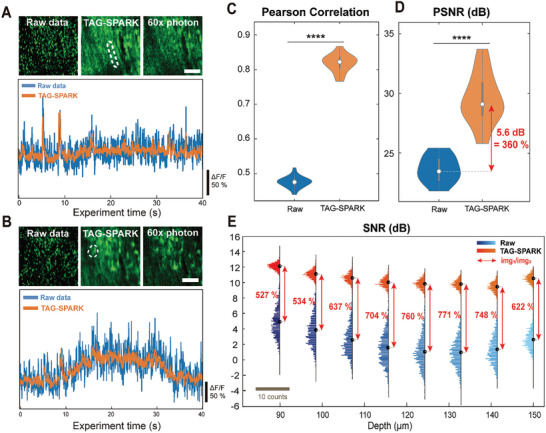
TAG‐SPARK denoising performance on calcium imaging of PCs. (A, B) (Top) Calcium imaging of PCs in the dendritic and somatic layers, respectively. From left to right: raw data, TAG‐SPARK denoised image, average across 60 different time points (60× photons). (Bottom) Corresponding calcium traces of a selected dendrite and soma within a 40‐s observation time window. C) Violin plots of the Pearson correlation between volumetric images of the raw and TAG‐SPARK denoised images with respect to the 60× photons average. D) Distribution of PSNR before and after TAG‐SPARK processing. The average value indicates a PSNR difference of 5.6 dB, corresponding to around 360% improvement in image quality between the 2 volumetric images. E) SNR enhancement of TAG‐SPARK among different depths of 2D layers, the best approaching 800%.

To validate the effectiveness and robustness of the TAG‐SPARK denoising algorithm, we assessed its performance on experimentally acquired two‐photon data of PCs in both structural (spatial) and functional (temporal) manners as shown in **Figure** **2**. The top panels of Figure 2A,B show the calcium snapshot at the dendritic and somatic layers, respectively. The raw images exhibit a stochastic signal distribution, resulting in indiscernible dendritic and somatic structures. Through applying the TAG‐SPARK denoising algorithm to improve the SNR, the resulting images unveil the biological context, where both dendrite and soma neuronal structures were capable of being segmented directly (white dashed line in Figure 2A,B). Moreover, the denoised image has much better image quality, compared to a moving average of N times photon images. The 60‐times averaged image, i.e., 60x photons image, achieved 90% PCC and reasonably high PSNR compared to the denoised image (see Figure S1, Supporting Information). The capability to reconstruct neuronal structures with two order‐of‐magnitude fewer photons manifests the potential of TAG‐SPARK for high‐speed imaging. While TAG‐SPARK is trained in a spatial manner, it preserves temporal information with the benefit of noise reduction in the temporal domain. The bottom panels of Figure 2A,B present the corresponding calcium traces of a selected dendrite and soma (white dashed line), where the denoised results (orange curve) present much‐reduced signal fluctuations. The main functional characteristics, including dendritic spikes and somatic slow responses, are well retained after the processing.

To quantitively evaluate the TAG‐SPARK performance, we computed the Pearson's correlation coefficient (PCC) of the raw and denoised functional volumetric image with respect to the 60x photons image at each time point. In Figure 2C, the violin plots depict that denoised images exhibited a much higher PCC distribution with a median of 0.81 in comparison to the raw data of 0.48. The elevated positive PCC value indicates that the denoising process has successfully preserved the neuronal structures and features. In addition, to present the improvement of overall 3D image quality and noise reduction, we calculate the distribution of the peak SNR (PSNR) before and after denoising. As shown in Figure 2D, the PSNR value increases by more than 5 dB, i.e., the effective noise reduction is over 300%. Both PCC and PSNR demonstrated statistically significant improvement, as evidenced by the results of the one‐tailed paired t‐test (p‐value < 0.001). To further confirm the effectiveness of noise reduction at various depths, Figure 2E presents a detailed 3D SNR analysis at different imaging depths, spaced at every 8 µm. The SNR values of raw data are between 1–5 dB (average 2.3 dB, blue color histograms), while those after TAG‐SPARK become 9–12 dB (average 10.4 dB, orange color histograms). The red double arrows and numbers indicate the ratio of SNR improvement of TAG‐SPARK, ranging from 7.2 dB (527%) to 8.9 dB (771%). On average, the overall SNR enhancement is 8.1 dB, corresponding to about 645% improvement in image quality. In addition, Figure S2 (Supporting Information) presents the depth‐dependent PCC and PSNR, further verifying the power of TAG‐SPARK for 3D image quality improvement. These findings underscore the efficacy of our approach in suppressing fluctuating noise in both spatial and temporal domains, while accurately maintaining the rapid neural activities observed in the calcium traces.

### 2.3 TAG‐SPARK Facilitates Calcium Dynamics Analysis of Extensive PCs Populations

**Figure 3:**
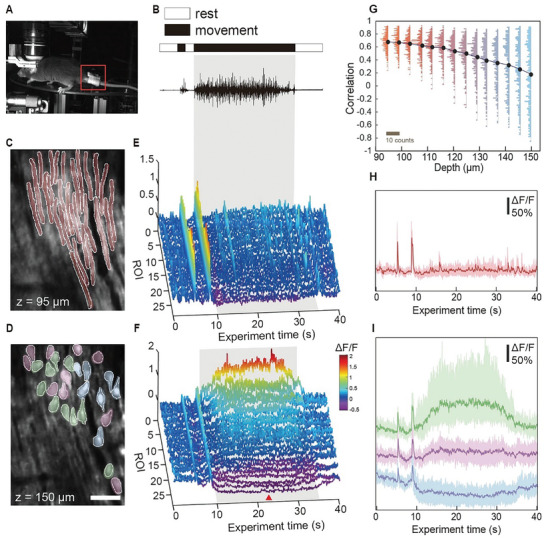
Calcium dynamics of PC populations at different depths. A) Photograph of the mouse head‐fixation and rotating disc system. The red square highlights the miniaturized three‐axis accelerometer installed at the base of the mouse's tail. B) Intensity profile (x‐axis) of the tail movements. C, D) Low‐speed scanning images in dendritic and somatic layers, respectively. E, F) Corresponding calcium traces of 27 cells, marked in (C) and (D). G) Correlation calculation at different depths over a 40‐s observation time window and the corresponding histogram distribution. H) Collective calcium response in the dendritic layers, showing all similar behaviors, marked in red. The same color is applied to the ROI markers in (C). I) Collective calcium response in the somatic layers. Three distinct behaviors are distinguished based on the threshold value of ΔF/F = 0.2 during the movement stimulation phase, represented by 3 different colors: green (ΔF/F > 0.2), purple (−0.2 < ΔF/F < 0.2), and blue (ΔF/F < −0.2). In (D), ROI markers are labeled with colors corresponding to their respective groups.

Leveraging the noise reduction capabilities of TAG‐SPARK in the spatiotemporal domain, we incorporated it into the examination of large populations of PCs in an awake mouse, aiming to explore volumetric calcium signal convolution from dendrite to soma at both the collective neuronal population and individual single‐cell levels. The theoretical expectation is that calcium influx in PC dendrites converges into the cell body, elevating the somatic calcium concentration necessary for the generation of complex spikes in PCs.^[^ ^15^, ^16^, ^17^ ^]^ However, the validation required high‐speed volumetric calcium imaging, is yet to be achieved. To validate this motion‐related calcium dynamics in PCs, we applied the volumetric imaging settings in a head‐fixed, awaken‐behaving mouse walking freely on a rotating disc (**Figure** **3**A). A miniaturized three‐axis accelerometer was installed at the base of the mouse's tail to quantify the mouse motion during the experiment (red square in Figure 3A). The accelerometer kinematics (Figure 3B; Figure S3, Supporting Information) were simultaneously recorded during calcium imaging. To better align the PC structure with calcium dynamics, we employed low‐speed scanning images to identify 3D cell morphology as structural masks. The denoised high‐speed scanning images facilitate the co‐registration process (Figure 5 in Materials and Methods). As a proof‐of‐concept experiment, 27 cells were extracted and marked in the red/green, blue, and purple shadows in the dendritic/somatic layers as shown in Figure 3C,D, respectively. The corresponding calcium traces of these PCs are depicted in Figure 3E,F. Consistent with previous studies, PCs exhibited similar dendritic calcium spikes during a long‐lasting movement marked with the gray area (Figure 3E). However, the calcium dynamics were divergent in PC cell bodies (Figure 3F). Some of them adhered to the conventional signal convolution theory, only different in the signal strength. Notably, others presented negative responses, as highlighted by the red arrowhead, inferring reduced calcium concentration that is against the common belief of additive convolution from dendrites to soma.

To further explore dendritic‐to‐somatic calcium evolution within PCs populations, we performed pairwise correlation comparisons of calcium traces among the 27 cells in each layer with 5‐µm depth intervals as shown in Figure 3G. From the top dendritic to bottom somatic layers, the averaged correlation coefficients decline from 0.7 to 0.1, and the histogram distribution illustrates the overall differences in each layer, particularly revealing more distinct variations in the deeper layers. At depths ranging from 95 to 115 µm, the correlation coefficient distributions were consistently positive, indicating similar calcium dynamics in the dendritic layers. However, beyond 120 µm depth, the distribution of the correlation coefficient spanned from positive to negative regions, suggesting the existence of at least three kinds of behaviors in the PCs populations in the somatic layers, i.e., positive, zero, and negative values of the correlation coefficients. The volumetric imaging results provided direct evidence that a significant portion of somatic PC responses do not follow their dendritic calcium activities. While imaging calcium activities of PC dendrites has been a standard approach to representing somatic complex spike activities of PCs, this discovery leads to a strong biological impact, and the dendritic‐to‐somatic interactions require revisiting. This phenomenon could only be accurately discerned through the simultaneous detection of dendritic calcium spikes and somatic calcium fluctuations facilitated by our volumetric imaging technique along with the contrast‐enhanced TAG‐SPARK algorithm.

For a comprehensive visualization of the overall calcium dynamics, Figure 3H,I illustrated the distribution of calcium traces for all 27 cells, displayed by colored shadows in the dendritic and somatic layers, respectively and the average calcium traces were represented by solid curves. In Figure 3H, all dendritic responses were averaged together, since they exhibited similar behaviors. On the contrary, in Figure 3I, three distinct behaviors in the somatic layer during the phase of the movement stimulation are classified based on the threshold value of ΔF/F = 0.2 (green: ΔF/F > 0.2, purple: −0.2 < ΔF/F < 0.2, and blue: ΔF/F < −0.2), showcasing the non‐intuitive convolution results (see Figure S4 (Supporting Information) for the raw data of all 27 cells). In Figure 3C,D, corresponding ROI markers are labeled with colors corresponding to their respective functional groups. An example of dendritic‐to‐somatic signal tracing along three individual neurons was given in Figure S5 (Supporting Information), with intensity contour maps to depict the complete relationship between observation depth and time.

Besides dendritic‐to‐somatic calcium evolution within PCs, the volumetric imaging technique also allows us to visualize the geometrical features between the three types of PCs based on their somatic responses (Figure 3D). The three types of PCs are geometrically mixed, indicating that spatial proximity is not the decisive factor in predicting the functional grouping of PCs. In brief, our volumetric imaging technique accompanied by the TAG‐SPARK denoising algorithm not only detects behavioral‐linked calcium dynamics in populational PCs but also identifies dendritic‐to‐somatic calcium propagations at a single‐cell level, revealing cell populations that deviate from the conventional knowledge of additive dendritic convolutions.

## 3 Discussion

In this study, we integrate a TAG‐lens‐based high‐speed volumetric system with a deep‐learning denoising algorithm, TAG‐SPARK, to demonstrate calcium functional imaging across a large population of PCs in awake mice. The continuously varifocal optics properties of the TAG lens realize dense oversampling in the axial scan at ≈200 kHz, leading to 265 layers in *z*, with 60 µm DOF to cover the PCs. Therefore, the gap between each frame is significantly smaller than the point spread function in the axial direction (see Materials and Methods). This capability effectively enhances spatial redundancy and reduces discontinuities among frames in volumetric images, facilitating effective training of the TAG‐SPARK model in the spatial domain. This ensures the preservation of temporal information in calcium dynamics and achieves over 300% PSNR and 700% SNR enhancement. To maintain animal safety and to enable long‐term volumetric functional inspection of neuronal dynamics, the excitation power is less than 100 mW (see Materials and Methods), which is well below the damage threshold of high‐speed 2PM when using a 80‐MHz Ti: sapphire laser.^[^ ^18^ ^]^ The utilization of TAG‐SPARK for image quality improvement eliminates the need for high‐power illumination, addressing concerns about photobleaching (Figure S6, Supporting Information). We present the applicability to neuroscience by examining calcium dynamics of PCs in the mouse cerebellar cortex, exploring signal transmission from dendrites to soma at various depths, and revealing variations among individual neurons.

For the TAG‐lens‐based high‐speed volumetric system, the axial scanning speed and DOF are primarily limited by the off‐the‐shelf TAG lens parameters, such as resonance frequency and diopter strength. As a resonant component, the TAG lens allows it to be driven in multiple harmonic frequencies. Higher driving frequency offers longer DOF extension but reduces the effective NA, resulting in lower spatial resolution.^[^ ^19^ ^]^ This tradeoff is considered in our experiment to fulfill critical requirements, including enough 1) temporal resolution to capture dendritic spikes, 2) spatial resolution to resolve sub‐cellular structure, 3) DOF to contain 3D neuronal architecture, and 4) dense z‐sampling dataset for TAG‐SPARK model training. In our experimental condition, we attain a 62 µm DOF to encompass the PC's neuronal architecture. Extending the DOF further is possible by increasing the TAG lens driving amplitude via adjusting its diopter strength. However, this leads to a decrease in the number of excitation photons per voxel, thus reducing the SNR that could impede model training. If a larger volume is required, apart from the deep‐learning denoising algorithm that we reported here, hardware integration such as adaptive optics (AO), that tackles deep‐tissue aberration, shall be beneficial to enhance SNR in 2PM, while maintaining the photon budget.^[^ ^20^ ^]^

TAG lens has been widely utilized in volumetric 2PM systems. One early milestone paper was in 2015,^[^ ^21^ ^]^ reporting 130 µm extension and 40 voxels in the z‐direction. Over the past decade, advancements in the application of 2PM with TAG lenses have substantially improved DOF extension and axial digital resolution. In 2018, Piazza et al. reported a DOF of 120 µm and axial digital resolution of 80 voxels,^[^ ^22^ ^]^ and Har‐Gil et al., achieved an impressive volume rate of 73.4 Hz, with each volume capturing a DOF of 330 µm using 150 voxels.^[^ ^23^ ^]^ In 2019, our group obtained functional imaging of fruit flies with a depth of 100 µm and 70 axial voxels.^[^ ^24^ ^]^ In 2021, the number became 200 µm DOF extension and 73 axial voxels, when we incorporated a GRIN lens endoscopy for volumetric functional inspection at 6 mm below the skull.^[^ ^25^ ^]^ In 2022, we integrated plug‐and‐play AO to address aberrations during high‐speed volumetric scanning.^[^ ^20a^ ^]^ However, these previous reports did not demonstrate sub‐micrometer axial layer gaps, which is necessary to implement the TAG‐SPARK idea. In 2023, Hsu et al. introduced a resonant galvo together with the TAG lens, pushing the numbers toward an axial resolution of 256 voxels, with a DOF of only 30 µm.^[^ ^26^ ^]^ Although the spatial redundancy should be adequate, their double‐resonant design resulted in Lissajous sampling, which might be problematic when combined with the Noise2Noise model. In this work, we demonstrated more than 200 axial layers full sampling with 60 µm in DOF extension, tailor‐made to realize the idea of deep‐learning denoise of TAG‐SPARK.

Except for the TAG lens, there are many other approaches for volumetric 2PM, but in general, the axial layer density is much less than the TAG lens approaches. For example, reverberation 2PM achieves video‐rate (30 Hz) observation across a DOF spanning 500 µm, but with a gap as large as 90 µm between each plane.^[^ ^27^ ^]^ Light bead microscopy (LBM), creates axially distinct foci at different depths simultaneously, enabling the direct acquisition of large volumetric images on a millimeter scale,^[^ ^28^ ^]^ but the layers are separated by at least 10 µm. Another recent multi‐plane imaging technique is dual‐objective 2PM, which offers a clear view of 380 µm thick brain slices with 50 axial layers; nevertheless, it is restricted to in vitro experiments due to the geometrical design.^[^ ^29^ ^]^

It is well known that image quality shall inversely depend on voxel dwelling time. In our 2P‐TAG lens system, this value is 0.02 µs per voxel, which may be further enhanced through spatiotemporal multiplex of laser beams. For example, LBM^[^ ^28^ ^]^ created 30 light beads in the axial direction, with 6.9 ns temporal delay between each bead; free‐space angular chirp enhanced delay (FACED) generated 80–100 foci in the lateral axis, with 2 ns delay among adjacent focal spots.^[^ ^30^ ^]^ It is interesting to note that they have reasonable contrast (or SNR) because they both utilized a high‐power (40–60 W), low repetition rate (1‐5 MHz), pump laser to drive an optical parametric amplifier as the laser source to fulfill the energy requirement of multiple beam splitting. Another reason is that their laser power after an objective lens was up to 450 mW, which is much larger than our 60–100 mW level. Nevertheless, the multiplex approaches require substantial hardware modification. In our case, we used a typical Ti:sapphire laser along with a single beam scanning, which is the most common design in 2PM setup worldwide. Our design involves minimal modification of the laser scanning system, with only a TAG lens and a corresponding 4f system, and the contrast enhancement comes from the TAG‐SPARK algorithm, not laser source engineering. We believe this is a much more cost‐effective solution for volumetric two‐photon microscopy, and we envision that our TAG‐SPARK algorithm applies to other volumetric imaging systems as long as the density in the axial direction is adequate to fulfill the spatial redundancy requirement.

While TAG‐SPARK follows the concept of Noise2Noise for noise reduction, our implementations differ significantly from the previous work. First, the conventional Noise2Noise model uses the same clean image with separately added random noise as input‐target pairs, whereas we use adjacent z‐frames to form these pairs. This approach is effectively similar to using slightly different clean images with added random noise. Thus, spatial continuity plays a vital role in TAG‐SPARK, which is facilitated by the TAG lens‐based high‐speed microscopy for its capability of acquiring dense z‐sampling at sub‐micrometer‐scale intervals. Next, unlike in Noise2Noise, where input‐target pairs are implicitly assumed to be independent and fed individually to the network model to learn noise reduction, we do not treat these pairs as independent. Instead, we stack all the input frames (red frames in Figure S7, Supporting Information) into a single 3D data stack and do the same for the target frames (yellow frames), creating two separate frame stacks for the model, as depicted in Figure S7 (Supporting Information). This enables us to leverage information across different frames with, for example, the 3 × 3 × 3 convolution kernels shown in Figure S7 (Supporting Information). Due to the 3D nature of these frame stacks, we utilize a 3D U‐Net for their processing, rather than the 2D U‐Net used in most Noise2Noise models. For instance, our 3D U‐Net model has fewer layers (25) compared to the conventional 2D U‐Net layers (34). Additionally, the sequence and the number of different types of layers vary between the two models (see comparison in Table S1, Supporting Information). The operations within the 3D U‐Net (e.g., convolution and pooling) are 3D‐based rather than 2D‐based. This allows us to exploit the information across different frames in the input and target stacks for effective noise reduction, as mentioned. Furthermore, the loss function we used is the average of the L1 and L2 norms to retain the advantages of both. The L1 norm enhances resilience to outliers, while the L2 norm promotes convergence during training. In contrast, Noise2Noise employs the L2 norm solely.

The advantage of TAG‐SPARK, as a spatial redundancy‐based algorithm, is to avoid spatiotemporal artifacts in 4D volumetric functional imaging. Here we compare TAG‐SPARK with several recently demonstrated deep‐learning models, including content‐aware image restoration (CARE), DeepCAD‐RT, and SUPPORT. The first one, CARE, is a “supervised” deep‐learning model that utilizes spatial information for training.^[^ ^31^ ^]^ Since CARE needs ground truth structural images as the training data source to improve the SNR, not a Noise2Noise model, we expect that CARE is superior in denoise performance. In Figure S8 (Supporting Information), we compare the SNR performance as well as the functional responses of raw data, CARE, DeepCAD‐RT, SUPPORT, and TAG‐SPARK, respectively. It is obvious that CARE offers the best SNR (Figure S8A,B, Supporting Information). Nevertheless, because the training focuses on structural information retrieval, CARE completely loses functional information, both fast and slow responses (Figure S8C, Supporting Information). Therefore, CARE is not suitable for reducing noise in dynamic neural signal imaging.

Then we compare TAG‐SPARK with DeepCAD‐RT,^[^ ^11^ ^]^ of which both are “self‐supervised” deep‐learning models. In Figure S8 (Supporting Information), we apply DeepCAD‐RT temporal redundancy‐based training to our data and compare it with training driven by spatial redundancy. The results manifest that TAG‐SPARK preserves rapid calcium dynamics and minimizes noise interference at non‐spiking time points, while DeepCAD‐RT dilutes a sharp signal peak and spreads artifact contamination to other frames, though it exhibits a better denoising effect. Regarding DeepCAD‐RT, if the functional response is not fast, such as the yellow area, it gives SNR improvement comparable to CARE, while keeping the functional observation. However, if fast spiking behavior is the target of interest, such as the red area and the inset in Figure S8C (Supporting Information), DeepCAD‐RT might create artifacts.

SUPPORT, as a remarkable “self‐supervised” deep‐learning model,^[^ ^13^ ^]^ is based on the insight that a pixel value in functional imaging is highly dependent on its spatiotemporal neighboring pixels. It uses a 2D U‐Net plus CNN to optimize the training result of xyt or xyz images, and in the published results, SUPPORT has demonstrated superior performance over DeepCAD‐RT, in particular the spike‐preservation capability, for xyt dataset. Nevertheless, the 2D training nature of SUPPORT limits its applicability to simultaneous 4D xyzt datasets, as manifested in Figure S8 (Supporting Information). When feeding the 4D dataset into SUPPORT, the spike in the functional response shifts its location, and additional noise is generated in the image. It is because the model cannot distinguish the image variations induced in time and in axial domains, resulting in reduced denoise efficacy, as well as artifacts of spatiotemporal functional responses. On the other hand, TAG‐SPARK is based on a 3D U‐Net model that is suitable to process the 4D dataset and gives the best balance between SNR and functional preservation. In addition, the complete volumetric time‐lapsed 4D data allows volumetric motion correction, which is one of the most critical problems for the functional inspection of living animals. In our case, since we captured a complete volume for functional imaging, motion artifacts can be corrected through the TAG‐SPARK pipeline, with its built‐in volume‐to‐volume motion correction, which enables the analysis of biological signals along the axial dimension. (Figure 5; Figure S10, Supporting Information)

In summary, when a transient response is critical, such as the fast‐spiking in PC dendrites, TAG‐SPARK is a better choice. On the other hand, for relatively slow dynamics, such as the convolutional result in PC soma, the temporal redundancy approach offers higher noise reduction. These two approaches coexist, with neither fully substituting the other. Thus, the mixture of temporal and spatial redundancies, as well as the corresponding deep‐learning denoising tools for different compartments of neurons, may generate optimal results with the best SNR for high‐speed 3D mapping. For example, in situations where images show spatial continuity without rapid temporal changes. In such cases, a new model combining the strengths of both approaches could potentially achieve better SNR. One straightforward approach is to first apply spatial denoising to the images, followed by temporal denoising for further SNR improvement. Another approach involves developing a new data stacking scheme to create xyzt image stacks as input and target stacks for a network model, such as a 4D U‐Net, to learn noise reduction. Therefore, further refinement of TAG‐SPARK is possible by integrating advanced network architectures, such as an attention mechanism.^[^ ^32^ ^]^

The strategic values of our volumetric system with the TAG‐SPARK algorithm are far beyond the realm of technical improvement, particularly in biomedical imaging. Besides the example of PCs, nearly all principal neurons and interneurons are intermixed to form microcircuits in 3D spaces. The knowledge of microcircuit interaction based on 2D technology is unable to explain critical biological functions, such as the day‐night cycle calculated by neurons in the suprachiasmatic nucleus,^[^ ^33^ ^]^ or the reward‐processing by the interaction between cholinergic interneurons and the medium spiny neurons in the striatum.^[^ ^34^ ^]^ High‐speed volumetric approaches with a contrast‐enhanced algorithm open a window to comprehensively study the populational interactions between neurons and their sub‐cellular calcium dynamics at the millimeter plus millisecond level.

## 4 Experimental Section

### Optical Setup

**Figure 4:**
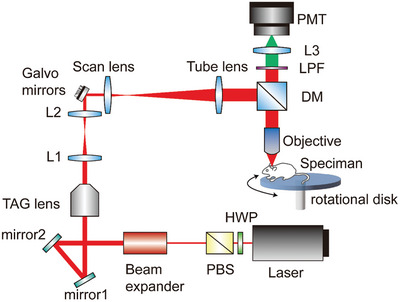
Schematic of home‐built high‐speed two‐photon volumetric imaging system. HWP: half‐wave plate; PBS: polarization beam splitter; TAG: tunable acoustic gradient; L: lens; DM: dichroic mirror; SPF: short‐pass filter; PMT: photomultiplier tube. Please refer to Materials and Methods for a detailed description of the optical setup.

**Figure** **4** illustrates the design of our home‐built high‐speed two‐photon volumetric imaging system driven by a tunable Ti:Sapphire oscillator (Chameleon Ultra II, Coherent). The excitation wavelength was tuned to 940 nm for two‐photon excited fluorescence of green fluorescent protein GCaMP6f. With a variable beam expander (#87‐564, Edmund), the laser beam size was adjusted to match the effective aperture of the tunable acoustic gradient lens (TAG lens 2.5β, TAG Optics). The laser beam was guided through a beam expander lens pair (AC254‐060‐B‐ML & LA1422‐B, Thorlabs), and then was relayed to a pair of galvanometric mirrors (6215H, Cambridge Technology) for 2D raster scanning. Through a telecentric scan lens and tube lens pair (SL50‐2P2 & TL200‐2P2, Thorlabs), the scanning pattern was reflected by a dichroic beam splitter (FF749‐SDi01‐25 × 36 × 3.0, Semrock) and directed onto the back aperture of a 25x objective (XLPLN25XWMP2, Olympus). To prevent laser damage and photobleaching in the mice, the power after the objective lens ranges from 60 to 100 mW. The emitted two‐photon fluorescence was epi‐collected by the same objective, passing through the dichroic beam splitter, and then focused by an imaging lens (LA4306‐A, Thorlabs) onto a photomultiplier tube (PMT) module (H14119‐40, Hamamatsu). Two bandpass filters (FF01‐520/15‐25, Semrock) were placed in front of the PMT to filter out the reflected excitation laser and undesired background signals. During the experiment, volumetric images were acquired in both low‐ and high‐speed scanning modalities. In the low‐speed scanning mode, the linear translation stage (MGZ30, Thorlabs) was employed to achieve axial translation with an axial spacing of 1 µm and a voxel dwelling time of 5 µs. Each frame was acquired after three times averaging, and the volume size of 400 × 400 × 152 (µm^3^), featuring 512 × 512 pixels per 2D image and 152 frames in the stack. On the other hand, the high‐speed scanning mode utilized a TAG lens for rapid z‐modulation at a resonant frequency of 188 kHz. The resulting volume dimensions were 200 × 400 × 62 (µm^3^) with a voxel dwelling time of 0.02 µs. The volume rate achieved was up to 23 Hz, with a 64 × 128 × 265 voxels image size.

To quantitatively determine the optical resolution of the 2P‐TAG microscopy system, we analyzed images of standard fluorophore beads with a size of 200 nm. We average a total of 85 measurements for full‐width half maximum (FWHM) values of lateral and axial profiles. The resulting resolution is 673.9 ± 0.52 nm and 4.96 ± 0.28 µm respectively, as depicted in Figure S9 (Supporting Information).

### Sample Preparation and Mice Movement Stimulation

This research employed adult male Pcp2‐GCaMP6f mice, produced by crossing Pcp2‐cre (Jackson Lab. #: 0 04146) with tTA2‐GCamP6 (Jackson Lab. #: 03 0328). The mice were housed in standard conditions with a 12‐h light/12‐h dark cycle and provided ad libitum food and water. Ethical approval was granted by the Institutional Animal Care and Use Committee of National Taiwan University (approval number NTU110‐EL‐00023). A surgical procedure was conducted to implant a cranial window on the cerebellar surface. Initially, a 4 × 4 mm piece of the skull was removed from the midline, 6 mm posterior from bregma. Subsequently, a 5 × 5 mm cover glass was placed on the cerebellar surface and affixed with glue to the skull. After the glue had healed, dental cement (Super‐Bond, Japan) was applied to the skull and the cranial window's edge. Finally, a head plate (Neurotar, US) was secured atop the cranial window using dental cement. The mouse underwent a one‐week recovery period before engaging in in vivo optical imaging experiments. During the experiments, the mouse was secured beneath the objective lens using a customized head‐fix plate and holder. A micromanipulator facilitated the movement of the holder to explore the brain window's region of interest. A transparent acrylic disc with a diameter of 30 cm was positioned beneath the mouse with its height adjusted to support the mouse in a posture conducive to normal walking. During the experiment, the mouse was initially kept in a stationary state and stimulated by slight back‐and‐forth movements of the disc to induce movement. The angular displacement of the disc was then detected by a rotary encoder and converted into a rotational speed signal by a myDAQ device and LabVIEW (National Instrument, US). Additionally, a miniature three‐axis accelerometer was mounted at the base of the mouse's tail to detect the mouse's motion during the experiment. For two‐photon imaging, low‐speed scanning was initially used to obtain 3D structural data. Then, the high‐speed scanning mode was utilized to record in vivo cerebellar GCaMP6f images for 40 s with or without the movement of the mice.

For each genetically labeled mouse, we gathered 12 independent datasets, and this procedure was repeated on three different mice to verify the practical applicability of our 2P‐TAG lens volumetric imaging technique.

### TAG‐SPARK Process

In accordance with the Noise2Noise concept, our proposed denoising process was designed to exploit the spatial redundancy inherent in TAG‐based image slices for effective noise reduction. To leverage the higher‐dimensional information (e.g., correlations across frames) in the 3D data stacks for noise reduction, we replaced the original 2D U‐Net with a 3D U‐Net. As Figure S7 (Supporting Information) illustrates, instead of feeding frame pairs individually, we stacked two separate frame stacks (red and yellow frames) as the input and target stacks to the model. Therefore, each set of adjacent z‐direction image slices was treated as independent samples that represent the same scene. These pairs served as input‐target pairs in the training of a 3D U‐Net model. The training process was elucidated by the following representation of the loss function:^[^ ^14^ ^]^

(1) $$\arg\min_{\theta }\sum_{i\;=\;1}^{K}L\left(f_{\theta }\left({\hat{x}}^{i}\right),{\hat{y}}^{i}\right),$$

where *L* signifies the loss function, while ${\hat{x}}^{i}$ and ${\hat{y}}^{i}$ denote samples obtained from the same scene but affected by independent instances of noise. In each epoch, the network model *f*_θ_ encountered different sets of noisy input and target samples to estimate the optimal parameters θ. The inclusion of multiple corrupted samples from the same scene positively influences the learning task. Despite sharing a common underlying structure, the mapping from one noisy sample to another exhibited variations. The integration of these diverse samples enriched the regression task, resulting in a more precise estimation of the target scene. To enhance the diversity of training samples, we introduced a random pairing swap with a 50% probability. The 3D U‐Net architecture includes the analysis path (contracting path) and the synthesis path (expansive path),^[^ ^35^ ^]^ as depicted in Figure S7 (Supporting Information). The analysis path involves convolutional and pooling layers to extract salient features while preserving the underlying image structure and reducing dimensionality through pooling. In the synthesis path, these features were integrated and up‐sampled to generate a clean version of the input image. Additionally, shortcut connections between unshrunk low‐level features from the analysis path and the synthesis path were incorporated to accelerate training convergence and strengthen image details.^[^ ^36^ ^]^ The operations within the 3D U‐Net (e.g., convolution and pooling) are 3D‐based rather than 2D‐based indicating an architectural adjustment designed to capture information in frame stacks more effectively. These modifications allow the 3D U‐Net model we used to have fewer layers (25) than the 2D U‐Net (34), as shown in Table S1 (Supporting Information). To formulate the loss function for our model, different from Noise2Noise which employed the L2 norm solely, we combined the advantages of both the L1 and L2 norms by taking their average. The L1 norm enhances resilience to outliers, while the L2 norm promotes convergence during training.^[^ ^37^ ^]^ Training the U‐Net architecture can be intricate, especially when segmenting images for batch training. However, in our specific scenario, each functional volumetric image was used to train the model without additional segmentation. The model was trained using PyTorch on a GPU computing platform equipped with a GeForce RTX 2080 Ti and 128 GB of DRAM. CUDA 11.1 was employed to harness the GPU's computational capabilities during training.

### Structural Mask and Co‐Registration of 2 Scanning Modalities

**Figure 5:**
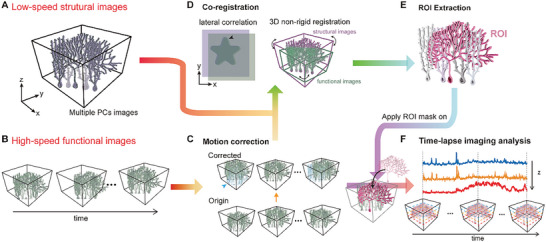
Schematic of cell segmentation and co‐registration of calcium imaging via low‐speed structural and high‐speed functional scanning modalities. A) Low‐speed scan images serve as the structural image. B) High‐speed functional images after TAG‐SPARK processing. C) Motion correction on functional images at different time points to ensure that the structures are aligned at the same position. D) Co‐registration of low‐speed structural and high‐speed functional images using lateral correlation analysis and 3D non‐rigid registration to identify the best correspondence. E) Segmentation of individual neurons using the structural images as ROI masks. F) Applying the ROI masks to time‐lapsed functional images and analyzing signal changes at different time points to capture calcium dynamic responses on a 3D basis.

Our imaging system coupled with the TAG‐SPARK denoising algorithm was capable of capturing the calcium imaging of neuronal populations with adequate SNR. For identifying single neuron behaviors, we integrated information from low‐speed scanning images, treated as structural masks, and high‐speed images, providing time‐lapse data for functional analysis (**Figure** **5**). The resultant denoised high‐speed scanning images and low‐speed scanning images underwent a series of steps for co‐registration. Initially, a 3‐s time projection was applied to the high‐speed scanning images. Subsequently, a rough manual alignment was performed through visual inspection to ensure both the high‐speed and low‐speed images were within the same field of view. For further refinement, the “Register Images” feature in the Amira software was utilized. This entailed configuring the high‐speed scanning volumetric images as the Model and the low‐speed scanning images as the Reference. A “Rigid” transformation with disabled rotation was selected, opting for “2D registration” with a threshold outside value of 0.8. The registration metric was “Normalized Mutual Information,” and the optimizer employed the “Extension direction”. Optimization involved varying the step size between 20 and 0.1, with the “finest value = 1” and a minimum “tolerance value = 0.0001.” These procedures ensure consistent alignment between the high‐speed scanning and low‐speed scanning images, enhancing overall reliability in data analysis and interpretation.

### Temporal Correction and Motion Correction of Functional Images

To conduct accurate time series analysis on calcium dynamics, it's crucial to correct for temporal offsets among voxels. This involves applying temporal correction, which rectifies voxel‐dependent delays. This correction is based on linear interpolation, shifting the time series of each voxel to align temporally with a reference time point.^[^ ^38^ ^]^ Moreover, during in vivo measurement, tissue movement would introduce unwanted fluctuations in recorded fluorescence signals, resulting in misinterpretation of calcium traces. To address lateral image position variations occurring at each time point due to tissue movement, lateral motion correction was applied to the denoised functional images. Assuming that head movement did not induce shear or scale transformation, a rigid body transformation using an affine transformation was employed. The transformation is expressed as follows:

(2) $$u\;=\;T_{\mathrm{moti}\mathrm{on}}w$$

where** **w** =  [*w_x_* *w_y_* *w_z_* 1]^*T*^ and** **u** =  [*u_x_* *u_y_* *u_z_* 1]^*T*^are the space coordinates of the functional images and the reference images, respectively. **T** *_motion_* is the transformation matrix

(3) $$T_{\mathrm{moti}\mathrm{on}}=T_{\mathrm{moti}\mathrm{on}}\left(t_{x},t_{y}\right)=\left[\begin{array}{cccc} 1 & 0 & t_{x} & 0\\ 0 & 1 & 0 & t_{y}\\ 0 & 0 & 1 & 0\\ 0 & 0 & 0 & 1 \end{array}\right]$$

where *t_x_* and *t_y_* specify the displacement along the *x*‐ and the *y*‐axis. The parameters in **T** *_motion_* were determined through an iterative optimization method:

(4) $${\hat{T}}_{\mathrm{moti}\mathrm{on}}=\;\arg\min_{T_{\mathrm{moti}\mathrm{on}}}C\left(T_{\mathrm{moti}\mathrm{on}},V_{\mathrm{func}\mathrm{tion}\mathrm{al}},\;V_{\mathrm{refe}\mathrm{rence}}\right)$$

where *C* denotes the similarity metric to be optimized during motion correction, which is the Mattes mutual information metric. **V** *_reference_* and **V** *_functional_* are the first functional and any one of the remaining functional scans, respectively. The optimization problem of Equation 4 was solved using the one‐plus‐one evolutionary optimizer.^[^ ^39^ ^]^

### Image Analysis

Low‐speed scanning images offer structural details, enabling the identification of individual neurons through 3D segmentation. The segmentation process employed the “Volume Edit” feature in the Amira software to precisely outline neurons in 3D space. Further refinement was achieved using the “Selection Brush Tool” in ImageJ, allowing the removal of background information outside cell boundaries in each layer. This combination of tools ensures accurate and detailed segmentation, facilitating subsequent analysis and characterization of neurons. For the analysis of specific layers, such as dendritic and somatic layers illustrated in Figure 3, 27 cells were individually chosen using the “Selection Brush Tool” in ImageJ to draw ROIs manually on 2D images. These ROIs served to create masks for extracting specific regions from high‐speed functional images. The MATLAB “dot product” function was then applied to retain only the signal within the designated ROIs, enabling temporal analysis of the selected areas. Calcium intensity analysis within each layer at different depths and time points was conducted by evaluating the integrated density of individual cells over time within each layer using MATLAB. This process provided a representation of calcium intensity for each layer at specific time points. Information from various depths and time points was systematically recorded to analyze temporal dynamics and calcium activity within the selected layers. To normalize the entire experimental period, the average fluorescence density *F* during the mouse rest state was utilized.

### Statistical Analysis Section

In Figure 2, the normalized intensity of each cell was obtained by dividing the average intensity during the mice's rest state. Pearson correlation coefficient (PCC) and peak signal‐to‐noise ratio (PSNR) are calculated across the entire 3D volume spanning 920 different time points. SNR is evaluated within 2D layers across 8 various depths, including all time points. Subsequently, the comparisons between the two groups of data (raw data and denoised data) were analyzed using a one‐tailed t‐test. The P values less than 0.05 indicate statistical significance (*p < 0.05; **p < 0.01; ***p < 0.005; and ****p < 0.001). In Figure 3G and Figure S2 (Supporting Information), the data analysis and visualization (histogram plot and violin plots) were using MATLAB. In Figure S9 (Supporting Information), data are presented as the mean ± standard error of 85 distinct point spread functions (PSFs) to calculate averaged lateral and axial resolutions. For animal experiments, we acquired 12 independent rest‐movement datasets from one mouse, and the volumetric contrast enhancement results were reproduced in 3 mice.

## Conflict of Interest

The authors declare no conflict of interest.

## Author Contributions

Y.‐T.H., K.‐C.J., J.‐C.L., and G.‐J.H. contributed equally to this work. M.‐K. P., S.‐C. W., and S.‐W. C. performed conceptualization. Y.‐T. H., K.‐C. J., C.‐L. C., and J.‐C. L. performed the investigation. Y.‐T. H., K.‐C. J., C.‐L. C., T.‐C. C., J.‐C. L., and B.‐C. C. performed methodology. Y.‐T. H. and K.‐C. J. performed visualization. M.‐K. P., S.‐C. W., and S.‐W. C. did supervision. Y.‐T. H., K.‐C. J., and G.‐J. H. Wrote the original draft. G.‐J. H., M.‐K. P., S.‐C. W., and S.‐W. C. wrote, reviewed, and edited the original draft. All authors revised and agreed to the final version of the manuscript.

## Supporting information

Supporting Information

## Data Availability

The data that support the findings of this study are available from the corresponding author upon reasonable request.
