# Supplementary material for: TAG‐SPARK: Empowering High‐Speed Volumetric Imaging With Deep Learning and Spatial Redundancy
Source: Adv Sci (Weinh). 2024 Sep 16;11(41):2405293. doi: 10.1002/advs.202405293 (PMC11892496; doi:10.1002/advs.202405293)
Supplement: Supplementary file 1 — Supporting Information [file ADVS-11-2405293-s001.pdf]

ADVANCED SCIENCE
Open Access

Supporting Information

for *Adv. Sci.*, DOI 10.1002/advs.202405293

TAG-SPARK: Empowering High-Speed Volumetric Imaging With Deep Learning and Spatial Redundancy

Yin-Tzu Hsieh, Kai-Chun Jhan, Jye-Chang Lee, Guan-Jie Huang, Chang-Ling Chung, Wun-Ci Chen, Ting-Chen Chang, Bi-Chang Chen, Ming-Kai Pan, Shun-Chi Wu* and Shi-Wei Chu**

Supporting Information

TAG-SPARK: Empowering High-Speed Volumetric Imaging with Deep Learning and Spatial Redundancy

Yin-Tzu Hsieh[1†], Kai-Chun Jhan[2†], Jye-Chang Lee[3†], Guan-Jie Huang[4†], Chang-Ling Chung[4], Wun-Ci Chen[2], Ting-Chen Chang[4], Bi-Chang Chen[5], Ming-Kai Pan[3,6,7,8], Shun-Chi Wu[2,8]*, Shi-Wei Chu[3,4,8]**

*Corresponding author. Email: Ming-Kai Pan, emorymkpan1979@ntu.edu.tw; Shun-Chi Wu, shunchi.wu@mx.nthu.edu.tw; Shi-Wei Chu, swchu@phys.ntu.edu.tw

This PDF file includes:

Figure S1 to S10
Movies S1 to S3
Table 1

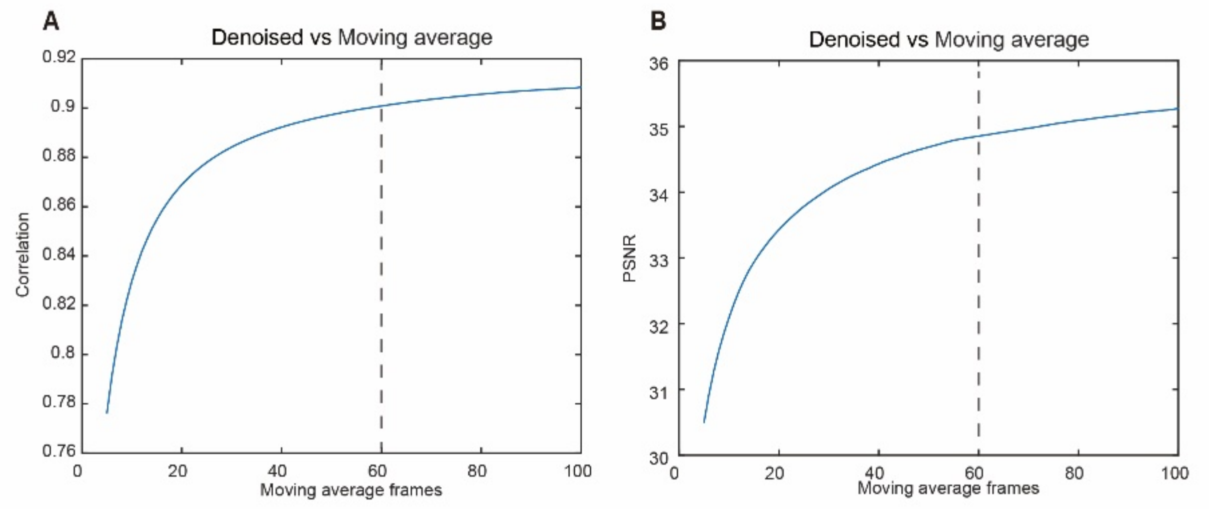

Figure. S1. Denoise and moving average comparison. (A) Pearson's correlation coefficient (PCC) and (B) peak signal-to-noise ratio (PSNR) of denoised images with respect to moving average of *N* times photons image. The 60-times averaged image, i.e. 60x photons image, has approached reasonably high PCC and PSNR versus the denoised image. That is, the quality of the denoised image is similar to that of the 60x average.

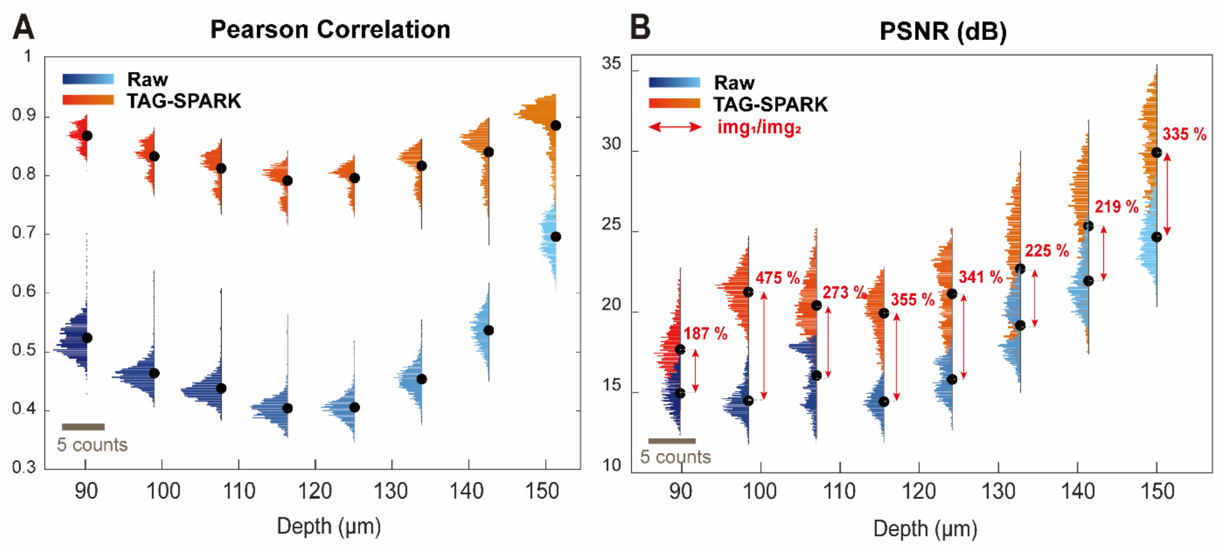

Figure. S2. TAG-SPARK denoising performance on 2D calcium imaging of PCs at different depths. (A) Pearson correlation and the corresponding histogram distribution at different depths before and after TAG-SPARK processing with respect to the 60x photons average. The PCCs of raw data range between 0.4-0.7, while those of TAG-SPARK are 0.8-0.9. (B) Distribution of PSNR and the corresponding histogram distribution at different depths before and after TAG-SPARK processing. The PSNRs of raw data range between 14-24 dB, while those of TAG-SPARK are 17-30 dB. The maximal enhancement is 475%.

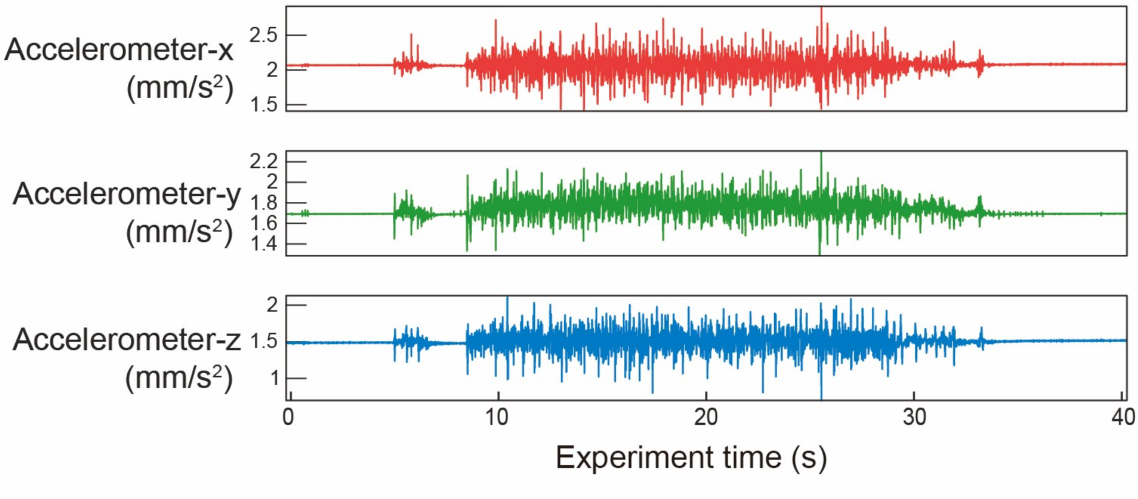

Figure. S3. Multiple-channel recording of the mouse movement. A three-axis accelerometer was attached to the mouse's tail to register its motion along the x, y, and z axes over the duration of the 40-second experiment.

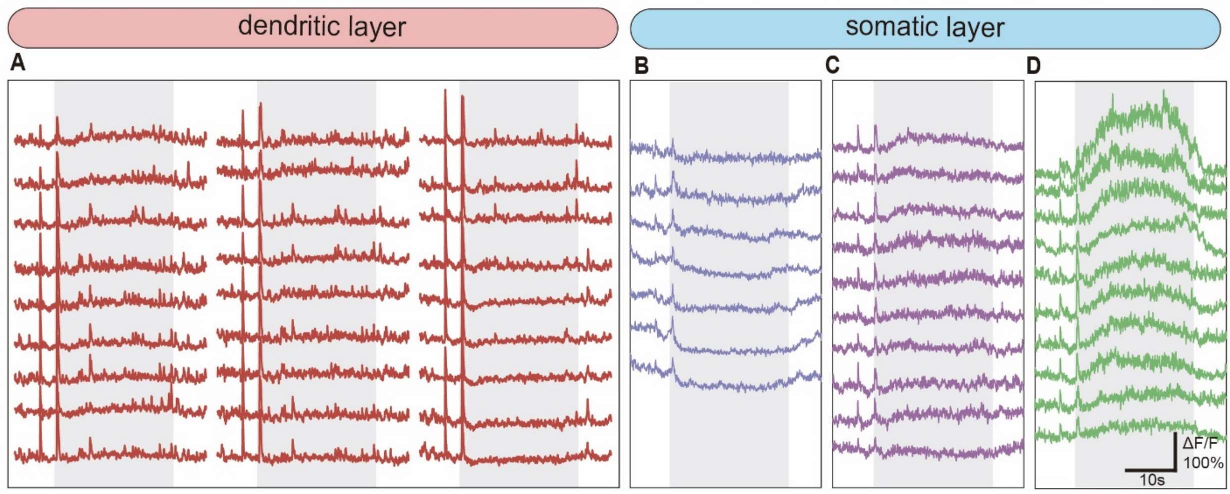

Figure. S4. Raw calcium traces for the 27 cells in the dendritic layer (A) and somatic layer (B-D). Three distinct behaviors in the somatic layer during the phase of the movement (gray shadow) are classified based on the threshold value of $\Delta F/F = 0.2$, (B) $\Delta F/F < 0.2$, (C) $-0.2 < \Delta F/F < 0.2$, and (D) $\Delta F/F > 0.2$.

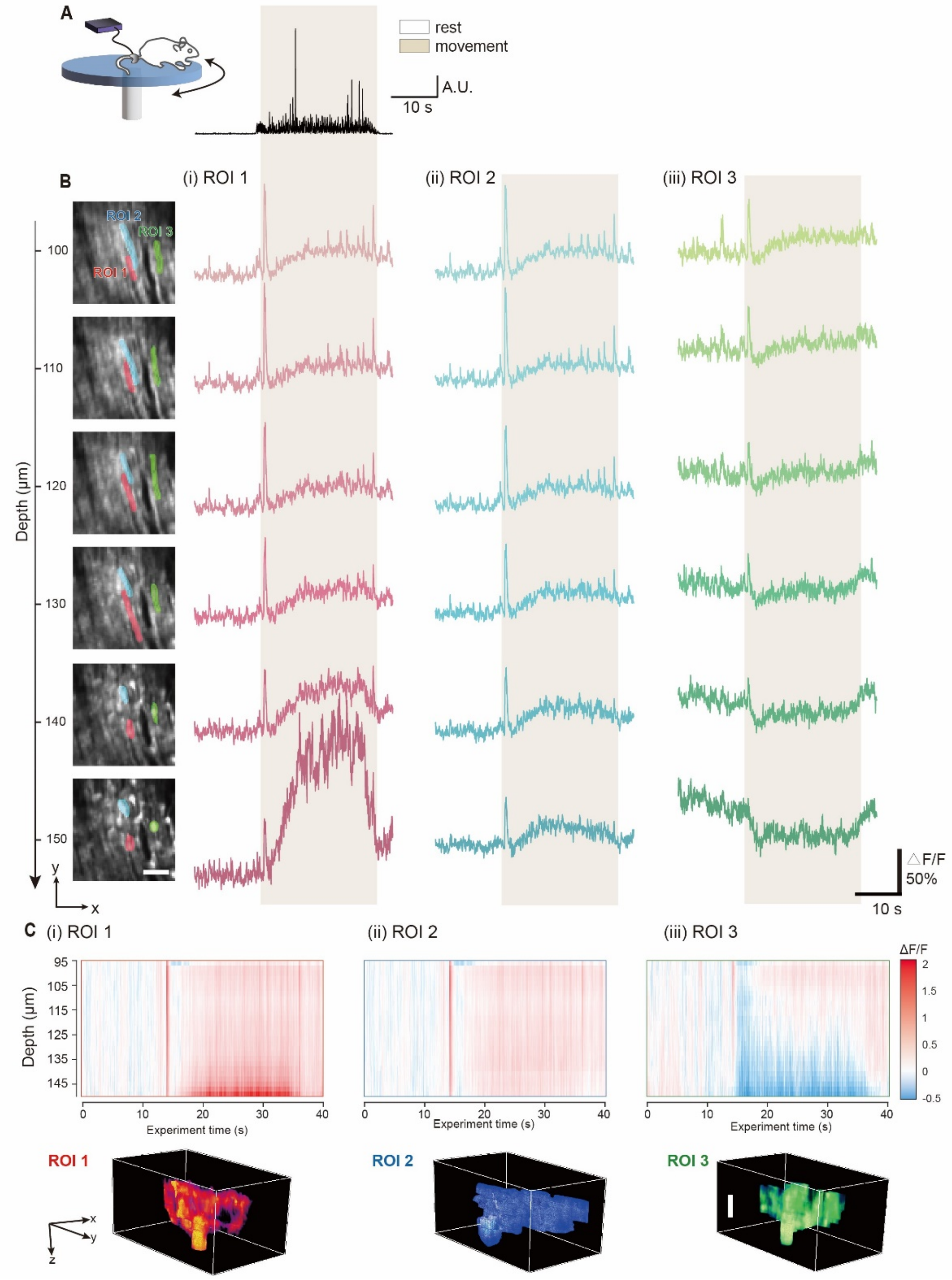

Figure. S5. Examples of dendrite-to-soma signal tracing along three individual neurons. (A) Schematic of a mouse placed on the rotating disc and the intensity profile of the leg movements. (B) (Left) Images of the mouse cortex of three individual PCs in different observation depths from 100 µm to 150 µm. The locations of three individual PCs are marked as ROI 1 (Red), ROI 2 (Blue), and ROI 3 (Green). Scale bar: 50 µm. (Right) Corresponding calcium traces at various depths. (C) (Top) Corresponding depth-dependent calcium response of three individual neurons. (Bottom) Three-dimensional image reconstruction of three individual neurons.

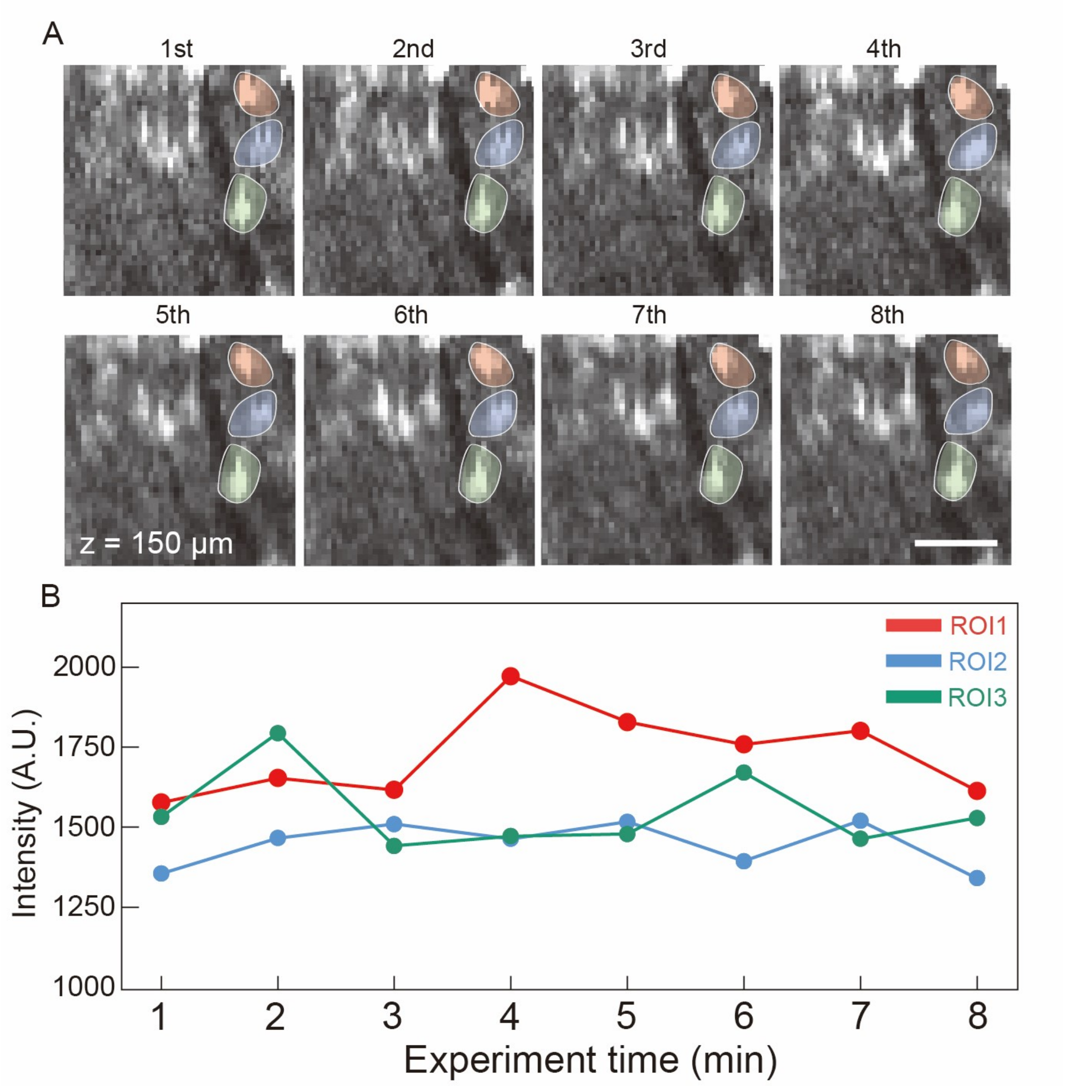

Figure. S6. Photobleaching examination during eight continuous high-speed volumetric imaging trials. (A) Raw calcium images of one optical section in the eight trials. (B) Average fluorescence intensity of the three ROIs across the eight trials, featuring that no significant photobleaching is observed.

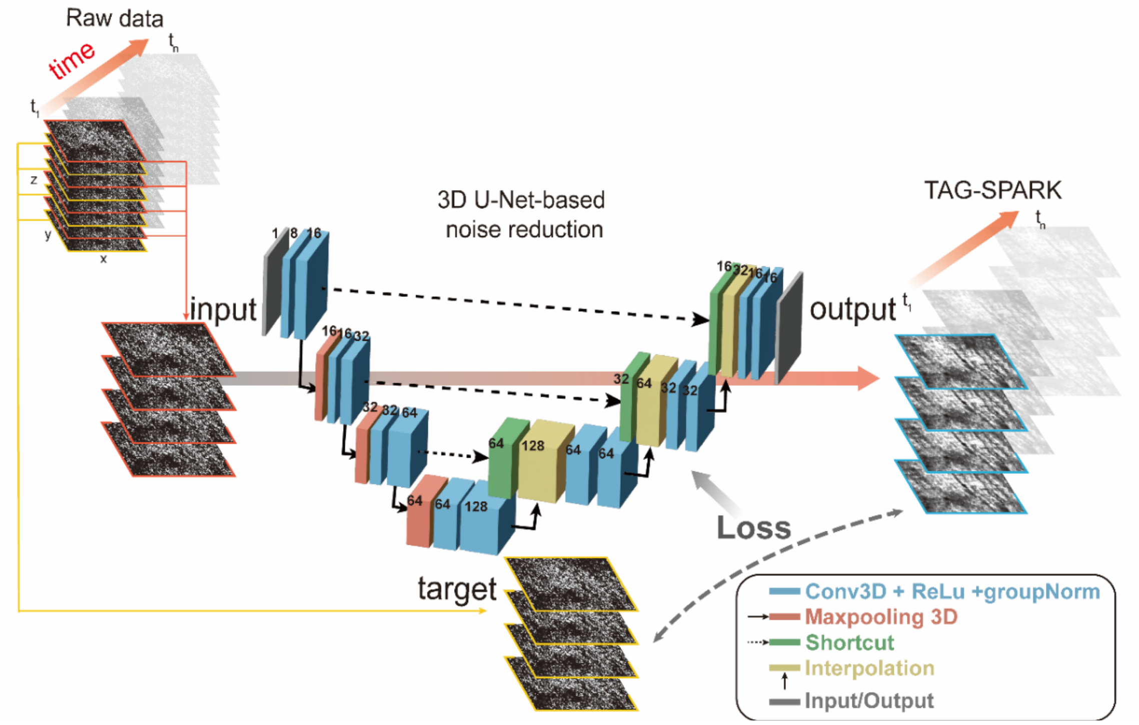

Figure. S7. Schematic of 3D U-Net model in TAG-SPARK denoising algorithms. TAG-SPARK utilized a 3D U-net model, which is composed of a 3D encoder module, a 3D decoder module, and skip connections from the encoder module to the decoder module. Multiple raw volumetric images at various time points are used for model training, where even layers (indicated by a yellow frame) and odd layers (indicated by a red frame) of each sub-stack are extracted to create two 3D tiles: one for input and the other for target volumes, which are used for training the network. The loss function is defined by comparing the output sub-stack (highlighted by a blue frame) with the target. Please refer to "TAG-SPARK process" in "Materials and Methods" for more details.

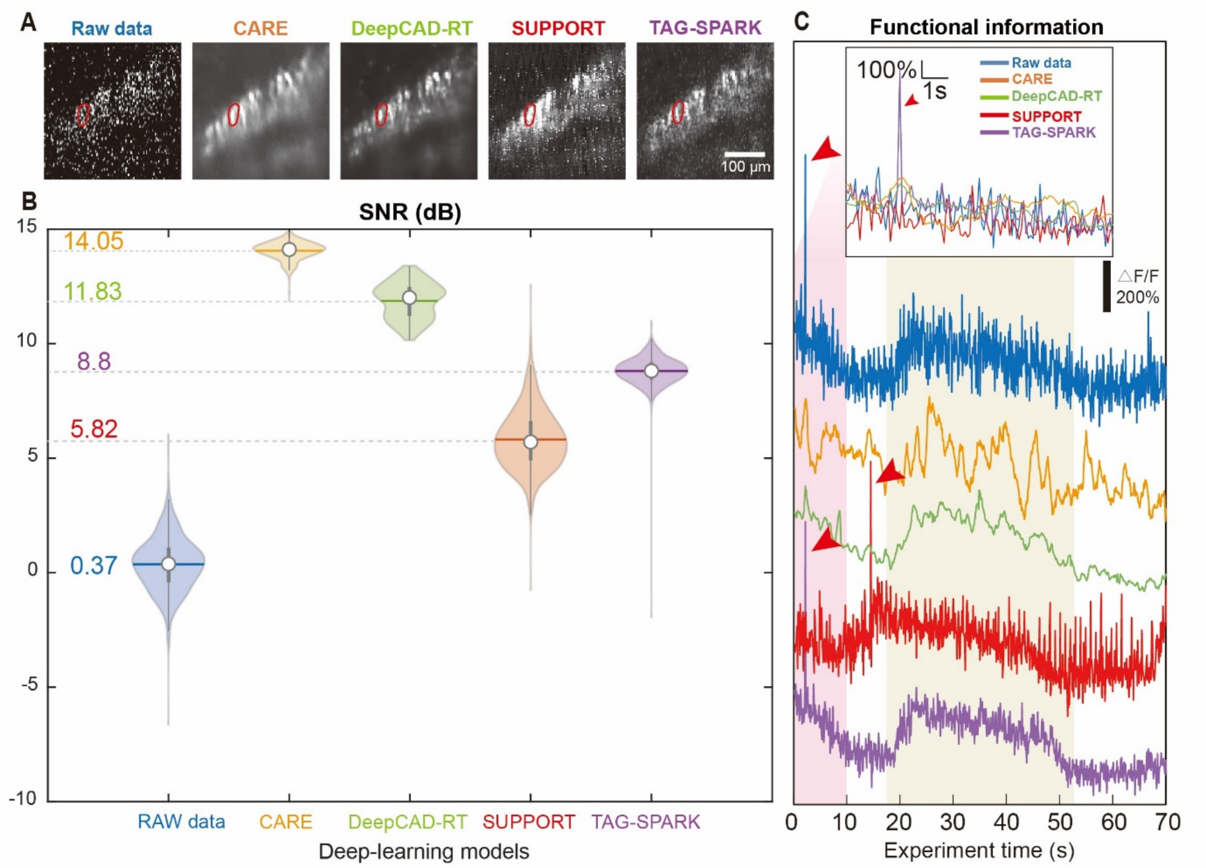

Figure. S8. Evaluation of different denoise deep-learning models on 2D calcium imaging of PCs. (A) *In vivo* calcium imaging of PCs: (Left to right) high-speed scanning image of raw data; denoised with CARE, based on spatial redundancy supervised learning; denoised with DeepCAD-RT algorithm, based on temporal redundancy self-supervised learning; denoised with SUPPORT algorithm, statistically unbiased prediction based on spatiotemporal information self-supervised learning; denoised with TAG-SPARK. Scale bar: 100 µm. (B) Corresponding SNR analysis shows that CARE and DeepCAD-RT provide comparable enhancements that are better than TAG-SPARK. (C) Calcium traces in an ROI encircle a bright neuron in (A), showing a slow peak during the 20-50s (yellow area), and a fast spike at 2s (red area). Although both CARE (orange curve), and DeepCAD-RT (green curve) seem to remove noise more efficiently, DeepCAD-RT smears the fast spike and CARE completely loses the functional information for both slow and fast responses. SUPPORT (red curve) maintains the trend of the signal but may also introduce artificial artifacts into the signal and shift the peak (red arrow). On the other hand, TAG-SPARK preserves all spike dynamics, with a reasonably enhanced SNR.

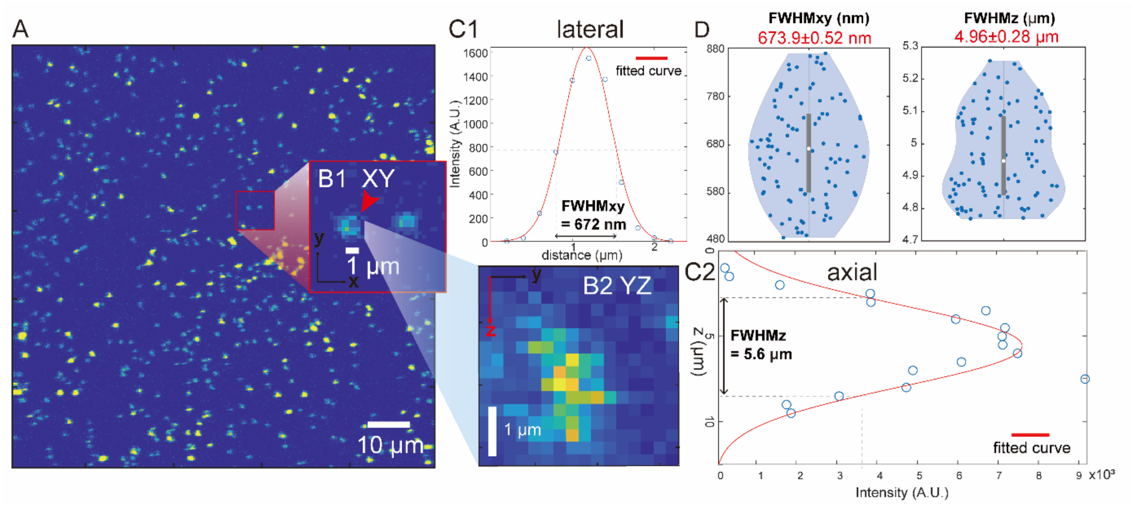

Figure. S9. Optical resolution. (A) A two-dimensional (2D) image of 200-nm beads. (B1) The XY image shows the red square region zoomed in from (A), with a single bead indicated by a red arrowhead. (B2) The YZ image of the same bead. (C1) The signal intensity profile shows that the lateral size of the bead is 672 nm. (C2) The signal profile demonstrates the axial size is 5.6 μm. (D) Average of 85 beads for FWHM values of lateral and axial profiles, indicating that the resolution is 673.9 ± 0.52 nm and 4.96 ± 0.28 μm, respectively. Please refer to the "Optical Setup" section in "Materials and Methods" for more details.

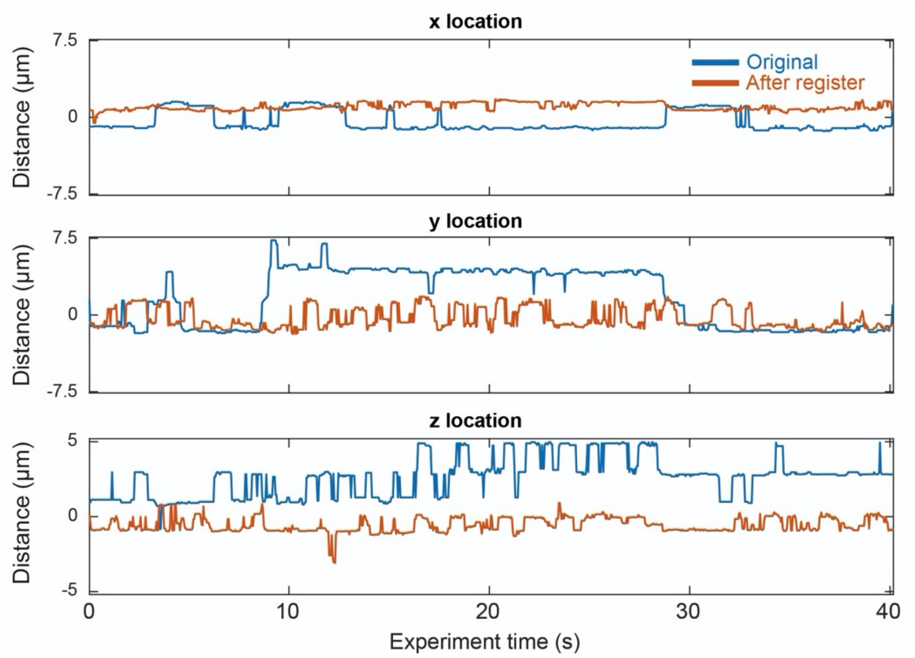

Figure. S10 Volume-to-volume 3D motion correction. Tracking the distance shift in three dimensions during motion correction on functional images at different time points. The blue curve shows the x-y-z location movement over time, while the orange curve represents the significantly reduced position shift, particularly the z direction, after applying motion correction across the entire time series.

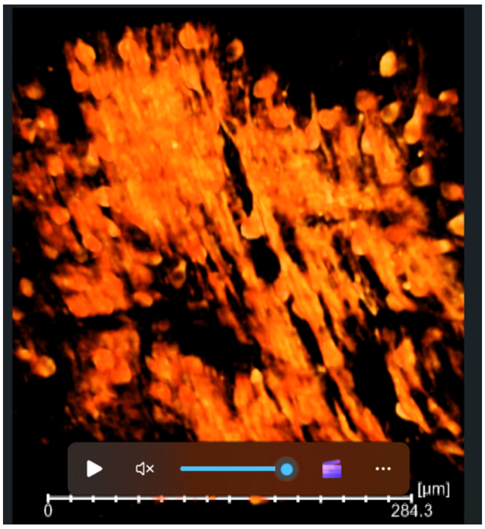

Movies S1. Three-dimensional structure of Purkinje cells.
Link:
https://drive.google.com/file/d/1k_s0oKCLsxqXxCLu8X3l4hynOEJCLcve/view?usp=drive_link

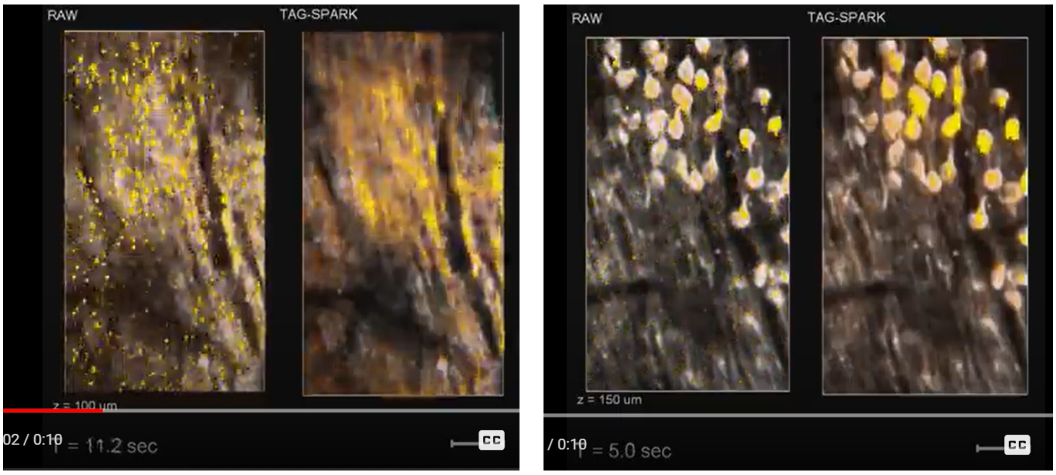

Movies S2. (a)(b) Functional imaging of Purkinje cells before and after TAG-SPARK denoising process in dendritic and somatic layers, respectively.

Link:
(a)https://drive.google.com/file/d/1u-N9qAynTQQJDclEKwW88ia9mQ9UTDmr/view?usp=drive_link

(b)
https://drive.google.com/file/d/1lX0Jgtt598XTTFmSGNxcBUBe3KRfhZ5T/view?usp=drive_link

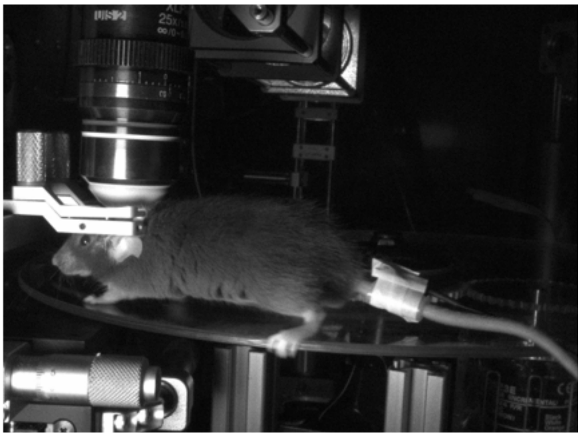

Movies S3. Mouse moving on the rotating disc system.
Link:
https://drive.google.com/file/d/1htwtLo1Jzv9ogfb17S6a1npB3DBjad0v/view?usp=drive_link

2D Noise2Noise Network Model			3D TAG-SPARK Network Model	
**NAME**	**FUNCTION**		**NAME**	**FUNCTION**
INPUT			INPUT	
ENC_CONV 0	Convolution 3 × 3		ENC_CONV3D 0	Convolution 3 × 3 × 3
ENC_CONV 1	Convolution 3 × 3		ENC_CONV3D 1	Convolution 3 × 3 × 3
POOL 1	Maxpool 2 × 2		POOL3D 1	Maxpool 2 × 2 × 2
ENC_CONV 2	Convolution 3 × 3		ENC_CONV3D 2A	Convolution 3 × 3 × 3
POOL 2	Maxpool 2 × 2		ENC_CONV3D 2B	Convolution 3 × 3 × 3
ENC_CONV 3	Convolution 3 × 3	ENCODE	POOL3D 2	Maxpool 2 × 2 × 2
POOL 3	Maxpool 2 × 2		ENC_CONV3D 3A	Convolution 3 × 3 × 3
ENC_CONV 4	Convolution 3 × 3		ENC_CONV3D 3B	Convolution 3 × 3 × 3
POOL 4	Maxpool 2 × 2		POOL3D 3	Maxpool 2 × 2 × 2
ENC_CONV 5	Convolution 3 × 3		ENC_CONV3D 4A	Convolution 3 × 3 × 3
POOL 5	Maxpool 2 × 2		ENC_CONV3D 4B	Convolution 3 × 3 × 3
ENC_CONV 6	Convolution 3 × 3			
UPSAMPLE 5	Upsample 2 × 2		INTERPOLATION 3	Upconvolution 2 × 2 × 2
CONCAT 5	Concatenate output of POOL4		SHORTCUT 3	Connect from ENC_CONV3D 3B
DEC_CONV 5A	Convolution 3 × 3		DEC_CONV3D 3A	Convolution 3 × 3 × 3
DEC_CONV 5B	Convolution 3 × 3		DEC_CONV3D 3B	Convolution 3 × 3 × 3
UPSAMPLE 4	Upsample 2 × 2		INTERPOLATION 2	Upconvolution 2 × 2 × 2
CONCAT 4	Concatenate output of POOL3		SHORTCUT 2	Connect from ENC_CONV3D 2B
DEC_CONV 4A	Convolution 3 × 3		DEC_CONV3D 2A	Convolution 3 × 3 × 3
DEC_CONV 4B	Convolution 3 × 3		DEC_CONV3D 2B	Convolution 3 × 3 × 3
UPSAMPLE 3	Upsample 2 × 2		INTERPOLATION 1	Upconvolution 2 × 2 × 2
CONCAT 3	Concatenate output of POOL2		SHORTCUT 1	Connect from ENC_CONV3D 1
DEC_CONV 3A	Convolution 3 × 3	DECODE	DEC_CONV3D 1A	Convolution 3 × 3 × 3
DEC_CONV 3B	Convolution 3 × 3		DEC_CONV3D 1B	Convolution 3 × 3 × 3
UPSAMPLE 2	Upsample 2 × 2		DEC_CONV3D 1C	Convolution 1 × 1 × 1, sigmoid
CONCAT 2	Concatenate output of POOL1			
DEC_CONV 2A	Convolution 3 × 3			
DEC_CONV 2B	Convolution 3 × 3			
UPSAMPLE 1	Upsample 2 ×			
CONCAT 1	Concatenate output of INPUT			
DEC_CONV 1A	Convolution 3 × 3			
DEC_CONV 1B	Convolution 3 × 3			
DEC_CONV 1C	Convolution 3 × 3, linear act.			

Table S1. Comparison of the architecture of Noise2Noise and TAG-SPARK network model. Please refer to “TAG-SPARK process” in “Materials and Methods” for more details.
